# Band‐Selective IR PRESS for Brain Tumor Spectroscopy Allows Robust Detection of Lactate

**DOI:** 10.1002/nbm.70220

**Published:** 2026-02-02

**Authors:** Shun Kishimoto, Daniel R. Crooks, Peng Lu, Yuki Shibata, Olga Kim, Jeeva Munasinghe, Otowa Yasunori, Yamashita Kota, Kazutoshi Yamamoto, W. Marston Linehan, Jing Wu, Murali C. Krishna, Jeffrey R. Brender

**Affiliations:** ^1^ Molecular Imaging Branch National Cancer Institute, National Institutes of Health Bethesda Maryland USA; ^2^ Urologic Oncology Branch, Center for Cancer Research National Cancer Institute, National Institutes of Health Bethesda Maryland USA; ^3^ Clinical Cancer Metabolism Facility, Center for Cancer Research National Cancer Institute, National Institutes of Health Bethesda Maryland USA; ^4^ Neuro‐Oncology Branch, Center for Cancer Research National Cancer Institute, National Institutes of Health Bethesda Maryland USA; ^5^ Laboratory of Functional and Molecular Imaging National Institute of Neurological Disorders and Stroke, National Institutes of Health Bethesda Maryland USA

**Keywords:** inversion recovery, lactate, lipid, magnetic resonance spectroscopy

## Abstract

Lactate plays a critical role in the tumor microenvironment, driving tumor progression, metastasis, and immune evasion. Despite its importance, in vivo quantification of lactate using magnetic resonance spectroscopy (MRS) has faced challenges, primarily due to the overlapping lipid signal at 1.3 ppm. Current clinical practice employs a long echo time to exploit differences in T2 relaxation between lactate and lipids; however, this approach significantly suppresses signals from other metabolites. Lipid has a notably different T1 relaxation time than lactate and other metabolites, which may be exploited by an inversion recovery sequence to better distinguish them. However, this method has not found wide use because of the loss of signal in other metabolites. Here, we introduce a selective inversion pulse with a short echo time MRS method (SPIR‐PRESS), which mitigates this issue. In phantom experiments, SPIR‐PRESS successfully suppressed lipid signals that could be misinterpreted as lactate in short TE PRESS spectra, while maintaining sensitivity to the full metabolite profile. SPIR‐PRESS demonstrated superior performance in quantifying lactate compared to long echo time PRESS, with ~60% increase in sensitivity for lactate detection compared to conventional PRESS with a 288‐ms TE. In a mouse glioma model, SPIR‐PRESS clearly detected lactate and other key tumor metabolites (total choline, creatine, NAA) in the tumor, which were not detectable in conventional long TE PRESS. These findings highlight SPIR‐PRESS as a promising technique for improved lactate quantification and comprehensive metabolite profiling in tumor environments.

## Graphical abstract

Lactate quantification by MRS is hindered by lipid signal overlap at 1.3 ppm, frequently forcing the use of long echo times that suppress other metabolites. We developed SPIR‐PRESS, a selective inversion recovery technique that suppresses lipids while preserving metabolite sensitivity. Phantom studies showed 60% improved lactate sensitivity versus standard 288 ms TE PRESS. In mouse gliomas, SPIR‐PRESS detected lactate alongside metabolites invisible in conventional long TE spectra. SPIR‐PRESS enables simultaneous lactate quantification and comprehensive metabolic profiling critical for tumor assessment.

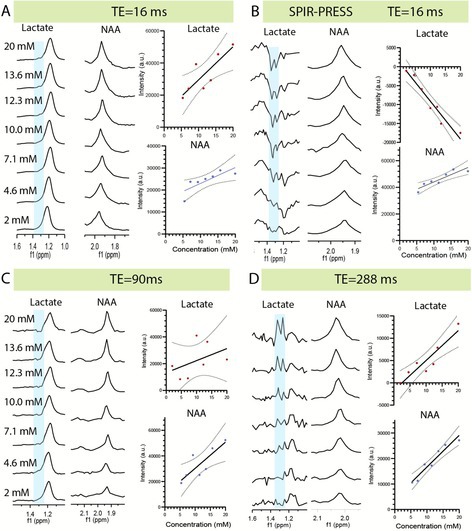

**Abbreviations**
CHESS: chemical shift selective
NAA: N‐acetyl aspartate
RARE: rapid acquisition with relaxation enhancement
SPIR‐PRESS: Spectral Presaturation with Inversion Recovery
ΔAICc: delta Akaike information criterion with correction for small sample sizes

## 1 Introduction

Emerging evidence suggests lactate is not only a passive byproduct of the Warburg effect, [1] but an active participant in immune resistance, metastasis, and tumor growth. Beyond its metabolic function, lactate acts as a multifaceted signaling molecule that drives cancer progression through multiple mechanisms. Lactate can be used as an alternative energy source for cancer cells, supporting their proliferation and survival [2, 3, 4]. Lactate also facilitates tumor cell migration and metastasis, contributing to cancer dissemination [5]. In the tumor microenvironment, it creates an acidic [6] radio‐ [7] and chemoresistant [8], immunosuppressive environment, enabling cancer cells to evade immune surveillance and contributing to resistance to therapeutic intervention across multiple treatment modalities. Consequently, lactate has been investigated as a potential biomarker for cancer diagnosis, prognosis, and treatment monitoring [9].

In clinical settings, lactate is routinely quantified in vivo using magnetic resonance spectroscopy (MRS). Despite the strong mechanistic rationale for its use, attempts to use the lactate signal from MRS as a prognostic biomarker for glioblastoma and other neoplasms have had mixed results [10]. While some studies have reported a positive correlation with tumor grade [11, 12], two large multicenter studies found only infrequent occurrence of lactate in long TE single‐voxel studies with no obvious correlation with tumor grade [13, 14]. This discrepancy arises from the ambiguous nature of the signal at 1.3 ppm [15]. The broad lipid signal at 1.3 ppm often dominates the short echo time spectrum of CNS tumors and healthy tissue of other organs. In the brain, the mobile triglyceride lipid signal arises from two sources: mobile lipids within the tumor [16, 17, 18, 19] and subcutaneous fat from the skull [20]. Several techniques can mitigate the signal from subcutaneous fat. One is outer volume suppression, which uses saturation bands during acquisition to nullify signals from outside the voxel of interest [21]. Another is k‐space spatial filtering, a postprocessing step that applies a filter to the k‐space data to modify the point spread function to suppress side‐lobe artifacts, thereby reducing signal leakage from the scalp into adjacent brain voxels [22]. However, the mobile lipids from micrometer‐sized lipid droplets that are common in CNS tumors cannot be easily removed by these techniques.

The most common method in clinical practice to distinguish the lactate peak from the overlapping broad lipid signal at 1.3 ppm exploits the faster transverse relaxation of lipids compared to lactate [20]. This is achieved by using a long echo time (TE) in the PRESS localization sequence [23]. At longer TEs, the lipid signal is rapidly attenuated while the lactate signal persists because of its longer T2 relaxation time. However, the use of a long TE comes at the cost of reduced overall signal‐to‐noise ratio (SNR) and suppression of signals from other metabolites with shorter T2 relaxation times. Typical TE values used for lactate detection are 144 or 288 ms. At TE = 144 ms, the lactate signal is expected to be inverted because of J‐coupling evolution. However, PRESS sequences can suffer from an artifact known as anomalous J‐modulation due to the finite bandwidth of the refocusing pulses that causes a spatial offset in the volumes selected for the methyl (1.33 ppm) and methine (4.1 ppm) protons [24]. This spatial offset means that in different parts of the voxel, the coupling partner can be either refocused incompletely or not at all, leading to significant signal cancellation. The effect is field dependent and is particularly severe at 3 T, where the large chemical shift difference between the protons approaches the pulse bandwidth [25]. Therefore, a TE of 288 ms is more commonly used to unambiguously detect the lactate peak [26], though at the expense of very low SNR where the lactate signal may be the only discernible metabolite resonance remaining. An ideal solution would be a method capable of resolving the lactate and lipid signals at a short TE, maintaining sensitivity to the full metabolite profile while avoiding excessive signal losses.

Several alternative approaches have been developed to separate lactate from lipid signals while preserving metabolite sensitivity. Despite these methodological advances, each approach presents limitations that restrict routine clinical implementation. Spectral editing techniques such as MEGA‐PRESS [27] and MEGA‐sLASER [28] utilize frequency‐selective editing pulses to differentiate lactate from overlapping lipids at short echo times [29, 30, 31]. While these methods maintain sensitivity to other metabolites, they rely on precise subtraction between subspectra and are therefore vulnerable to motion artifacts that can compromise signal cancellation [32]. Multiple quantum filtering (MQF) techniques suppress lipid contributions by selecting only coupled spin systems, effectively eliminating lipid contamination [33]. However, this approach also removes uncoupled metabolites such as N‐acetylaspartate (NAA), creatine (Cr), and total choline (Cho), which are valuable diagnostic biomarkers. Diffusion‐weighted spectroscopy exploits differences in molecular mobility to suppress lipid signals, but typically requires longer acquisition times and suffers from reduced SNR due to diffusion weighting [34]. An ideal solution would be a simple, fast method capable of resolving lactate and lipid signals at short TE while maintaining sensitivity to the full metabolite profile.

To overcome the limitations associated with existing techniques, the substantial difference in T1 relaxation times between free lipid and lactate can be leveraged by using an inversion pulse preceding voxel spectroscopy to distinguish between the two metabolites (SPIR: **S**pectral **P**resaturation with **I**nversion **R**ecovery). Our approach is similar to that described by Balchandani et al. for fat suppression in 1H MRSI at 7 T, although no rigorous quantification for lactate or comparison with other methods has been performed to our knowledge. By using a spectrally selective inversion pulse to minimize T1 relaxation of other metabolites, we show that the short echo SPIR‐PRESS significantly outperforms the standard long echo (144/288 ms) sequence used in clinical practice for lactate quantification and allows the quantification of other metabolites.

## 2 Methods

### 2.1 Animal Study

The orthotopic xenograft mouse glioma models were generated by intracranially injecting glioma cells into 8‐ to 12‐week‐old female immunodeficient mice [35]. In brief, NSG (NOD scid gamma) mice were anesthetized with a combination of xylazine (20 mg/mL) and ketamine (100 mg/mL) diluted in 0.9% injection saline at a 1:1:4 volume ratios for a total dose of 0.1 mL per 20 g body weight. After the animals were fully anesthetized, they were placed and immobilized in a small animal stereotactic frame fitted with a mouse‐specific headpiece using lidocaine gel pretreated ear bars. All surgery procedures were done under sterile conditions. The glioma cells were slowly injected intracranially (2 mm anterior and 2 mm lateral to bregma; 2.5 mm deep from the dura). The burr hole was closed with bone wax, and the scalp closed with Vetbond. Two models were made. For the glycolytic model, GBM1 cells (0.5 × 10^6^ cells/2 mL) were used to generate a glioblastoma model. For the nonglycolytic PDX model [36], a cell suspension of 1 × 10^6^ cells suspended in 10 uL of PBS from an oligodendroglioma grade III IDH1 mutant specimen was used. All animal experiments were approved by the National Cancer Institute Animal Care and Use Committee (NCI ACUC) and conducted in accordance with NCI ACUC guidelines under the authority of the animal protocol (NOB‐023).

UOK161 clear cell renal cell carcinoma (ccRCC) tumor xenografts were generated by injecting 5 × 106 cells subcutaneously into the hind leg of 10‐week‐old female NSG mice (Charles River) as cell suspensions in 100% Matrigel (BD Biosciences, San Jose, CA, USA). Animal experiments with UOK161 were approved by the National Cancer Institute Animal Care and Use Committee (NCI ACUC) and conducted in accordance with NCI ACUC guidelines under the authority of the animal protocol (PB‐029).

Animals were observed frequently while they recovered from the anesthesia in a warm, draft‐free area. During anesthesia, a pressure transducer (SA Instruments Inc.) was used to monitor the respiratory rate, which was maintained at 60 ± 10 breaths per minute. A nonmagnetic rectal temperature probe (FISO) was used to monitor the core body temperature, which was maintained at 36 ± 1°C using a circulating water‐warming pad.

T2‐weighted MRI images were acquired using a 3 T Biospec MRI scanner with a Rapid Acquisition with Relaxation Enhancement (RARE) pulse sequence. The following parameters were used: repetition time (TR) = 2366 ms, echo train length = 8, echo time (TE) = 48 ms, and number of averages = 4. The imaging volume consisted of 20 slices, each with a size of 2 cm × 2 cm and a thickness of 3 mm.

### 2.2 Magnetic Resonance Spectroscopy

#### 2.2.1 Instrumentation

All MRS experiments were conducted on a 3.0 T Bruker Biospec animal scanner (Bruker, Billerica, MA, USA; software version: Paravision V2.0) equipped with a B‐GA105S HP‐gradient system. Two different radiofrequency coils were used depending on the study. For all in vivo mouse brain studies, a standard Bruker 2‐channel ^1^H quadrature transmit/receive coil was used. For all in vitro phantom studies and in vivo mouse leg xenograft studies, a home‐built ^13^C‐^1^H dual‐tuned transceiver coil was used. This coil consisted of two 47 mm long, crossed saddle coils with a 31‐mm inner diameter, constructed from silver‐plated, insulated, multistranded copper wires to maximize the quality factor (Q) [37]. For all studies, voxel‐specific shimming was performed using an iterative, second‐order FASTMAP routine to optimize magnetic field homogeneity [38], with an acceptance threshold of < 15 Hz for the unsuppressed water linewidth. The acquisition window was set to capture the free induction decay corresponding to the right half of the final spin echo.

#### 2.2.2 In Vitro Phantom Studies

**FIGURE 1:**
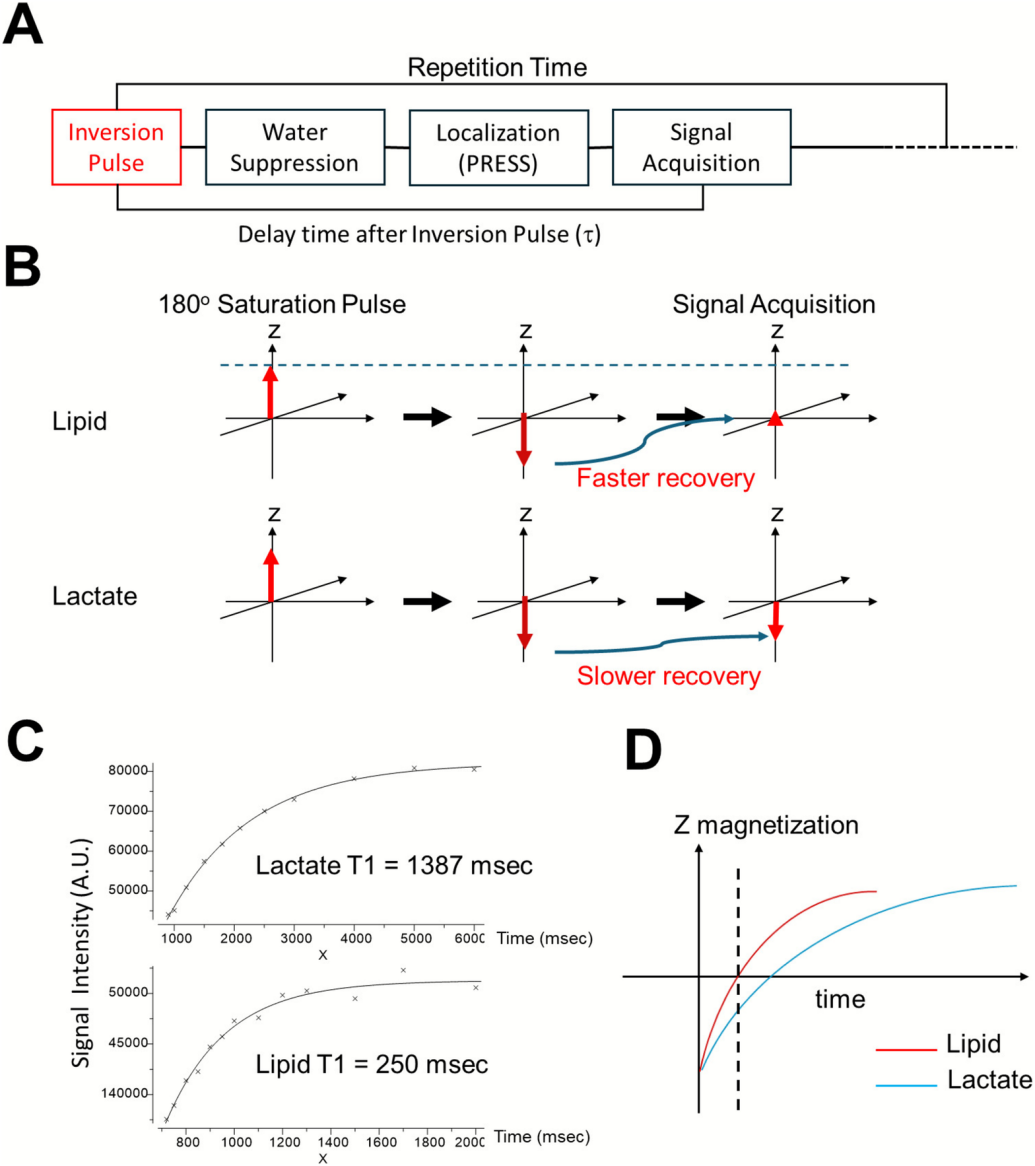
(A) SPIR‐PRESS pulse sequence with an inversion pulse and delay τ preceding PRESS localization, water suppression, and acquisition. (B) Schematic magnetization recovery showing selective nulling of the lipid signal at τ while lactate remains nonzero. (C) Inversion‐recovery data giving T1 ≈ 1387 ms for 111‐mM lactate and ≈ 250 ms for 4% lipid. (D) Schematic timeline summarizing the lipid‐null point relative to lactate recovery.

**FIGURE 2:**
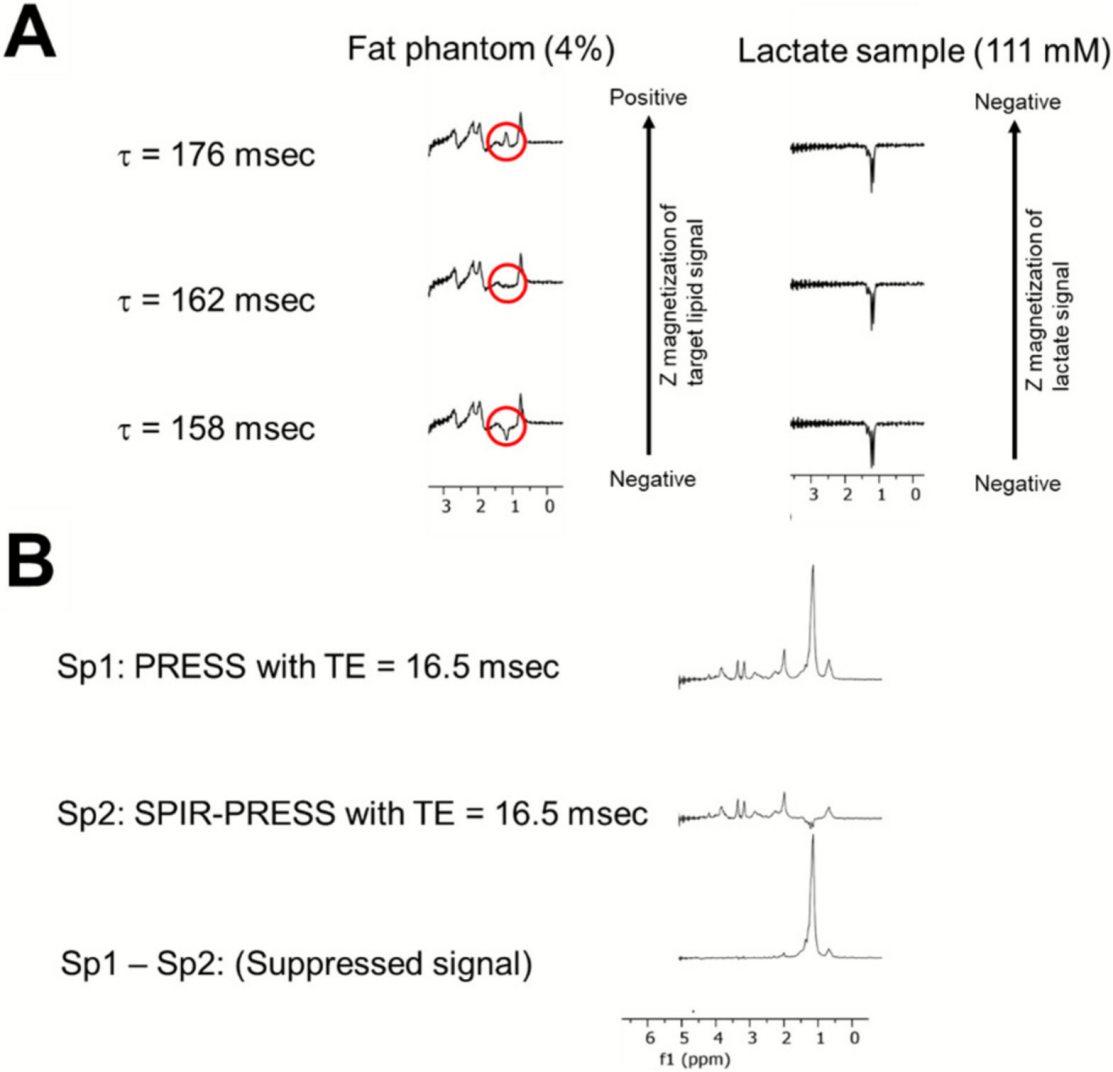
(A) Optimal null point of 4% Intralipid sample. (B) 16.5‐ms TE PRESS spectrum of a 4% Intralipid/111 mM lactate sample (top) along with the corresponding SPIR‐PRESS (middle) and difference spectra (bottom).

**FIGURE 3:**
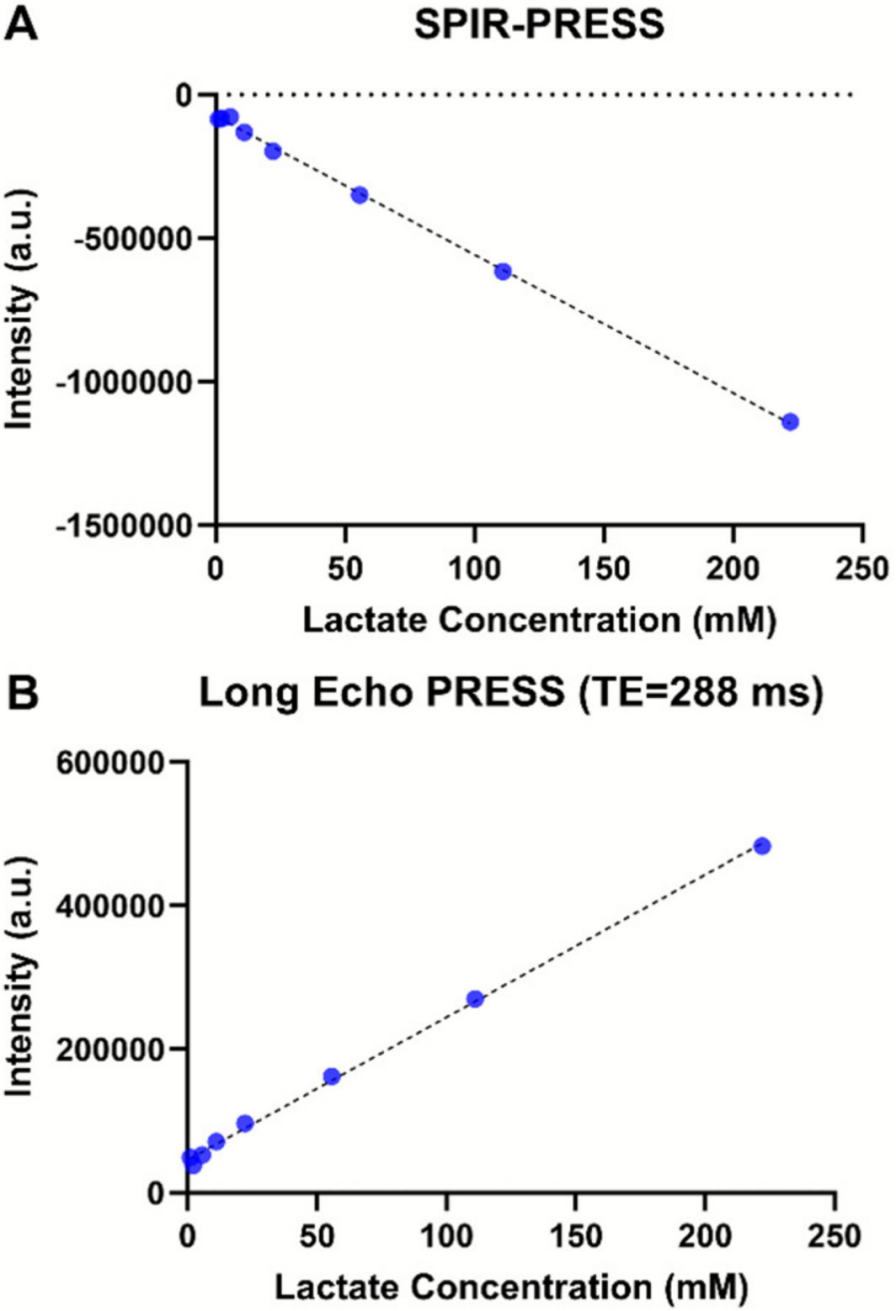
Calibration of lactate peak intensity versus concentration in 4% Intralipid phantoms: (A) SPIR‐PRESS (TE = 16.5 ms); (B) long‐TE PRESS (TE = 288 ms). At high SNR, both calibrations are highly linear, with a larger slope for SPIR‐PRESS.

**FIGURE 4:**
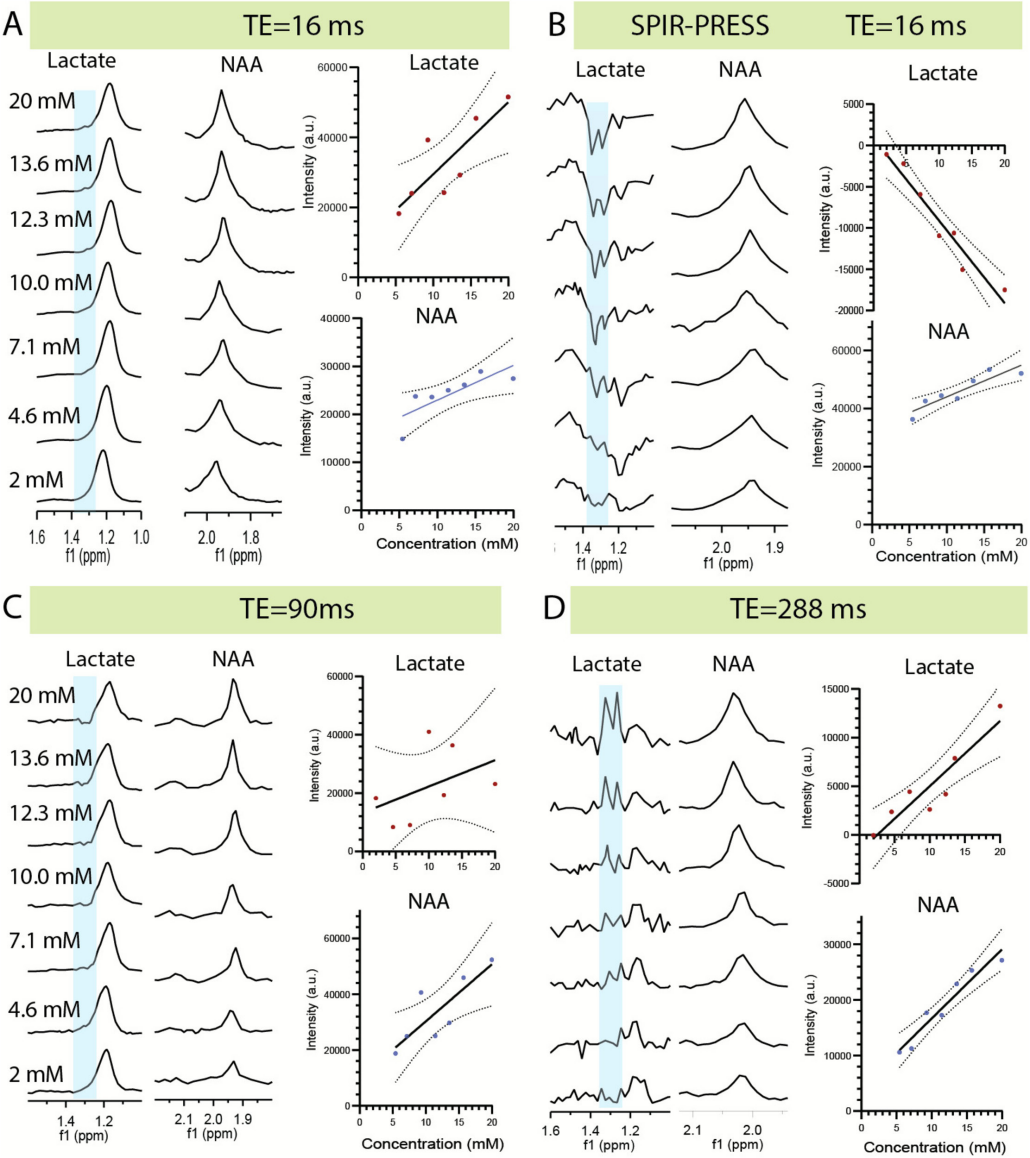
Quantification of lactate and N‐acetylaspartate (NAA) in mixed‐metabolite phantoms with lactate concentrations from 2–20 mM as indicated. Concentrations of other metabolites are listed in Supplementary Table 1; lipid was held at a constant 1:1 M ratio to lactate for each sample. Panels compare spectra acquired with (A) short‐TE PRESS (TE = 16 ms), (B) SPIR‐PRESS (TE = 16.5 ms), (C) intermediate‐TE PRESS (TE = 90 ms), and (D) long‐TE PRESS (TE = 288 ms). Blue shading indicates the integration window for the lactate doublet (~1.33 ppm), which is phase‐inverted in the SPIR‐PRESS spectrum (B). Adjacent plots show linear regressions of integrated signal versus nominal concentration (solid lines) with 95% confidence bands (dotted lines). SPIR‐PRESS yields a steeper lactate calibration slope and higher linearity than long‐TE PRESS, while NAA quantification remains comparable between the methods.

**FIGURE 5:**
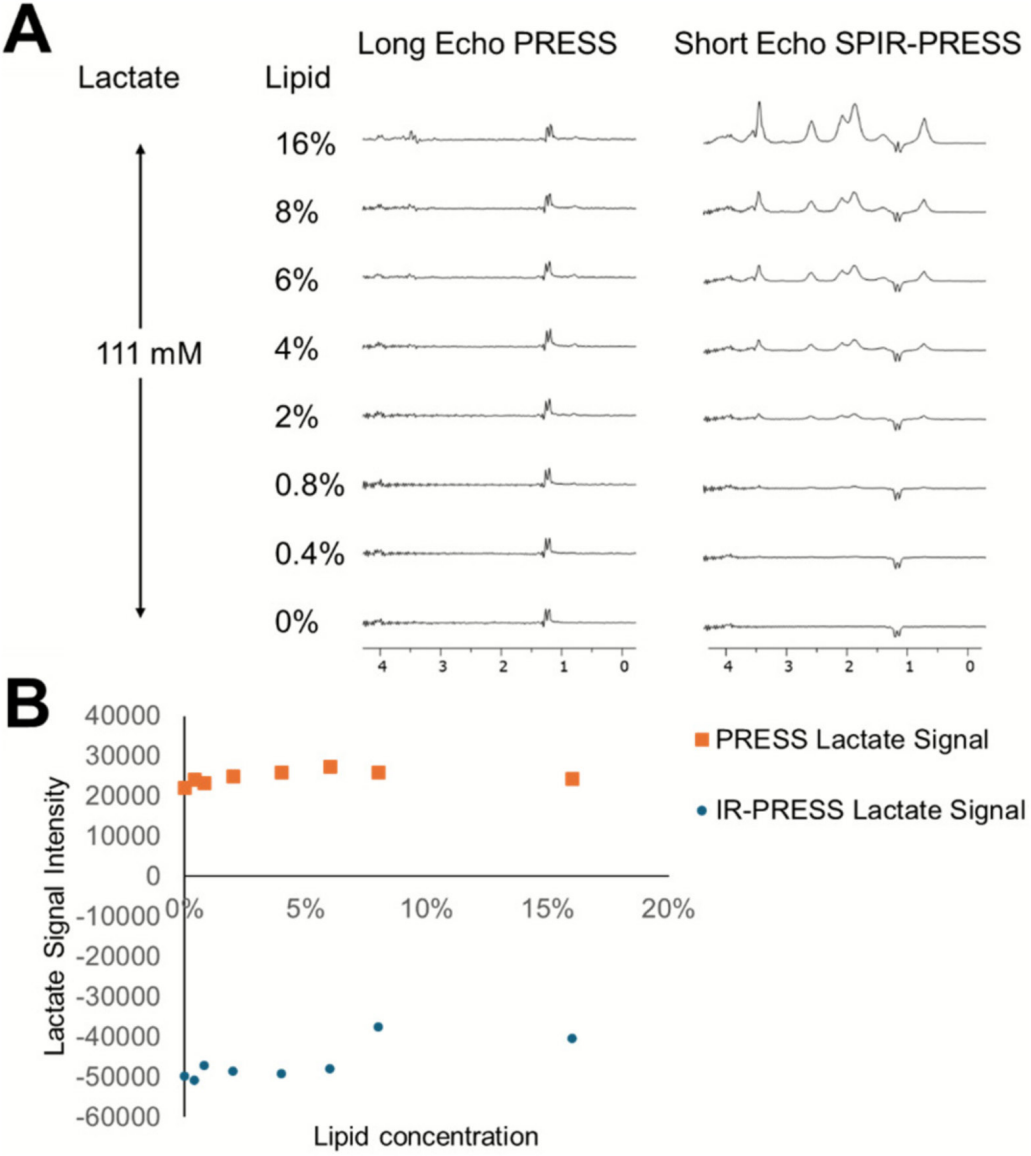
(A) Long‐TE PRESS (TE = 288 ms) and short‐TE SPIR‐PRESS (TE = 16.5 ms) spectra of 111 mM lactate acquired with increasing lipid content. (B) Corresponding lactate peak intensities versus lipid percentage. SPIR‐PRESS provides a stronger lactate signal but becomes susceptible to lipid‐driven baseline shifts when the lipid/lactate molar ratio exceeds about 6:1, whereas long‐TE PRESS remains stable across the tested range.

For all phantom studies, the pH of the aqueous solution was adjusted to 7.0 by titration with sodium hydroxide. To measure T1 relaxation times and optimize the inversion delay for lipid nulling (Figures 1C, 2), single‐component phantoms of 4% w/v lipid emulsion and 1% w/v lactate were scanned using an inversion recovery PRESS sequence (TR/TE = 7000/16.5 ms, 5 × 5 × 5 mm^3^ VOI, NA = 32). To compare the sensitivity of SPIR‐PRESS and long TE PRESS (Figure 3), phantoms containing 4% Intralipid and varying lactate concentrations were scanned using an 8 × 8 × 8‐mm^3^ VOI with either SPIR‐PRESS (TE = 16.5 ms) or long TE PRESS (TE = 288 ms). For all PRESS‐based acquisitions, 1.0‐ms hermitian refocusing pulses with a bandwidth of 3420 Hz were used. The SPIR‐PRESS sequence utilized an 18‐ms spectrally selective Gaussian inversion pulse (150‐Hz bandwidth) centered at 1.3 ppm. To evaluate performance in a complex metabolic environment (Figure 4), a series of mixed metabolite phantoms were prepared with concentrations of lactate (2–20 mM), NAA (5.4–20 mM), choline (1.35–5.35 mM), glutamine (1–5.0 mM), and creatine (6–9.6 mM). These were scanned with a 5 × 5 × 5‐mm^3^ VOI using SPIR‐PRESS (TE = 16.5 ms) and conventional PRESS at multiple echo times (16, 90, and 288 ms). To investigate the impact of varying lipid content (Figure 5), phantoms containing a fixed concentration of 111 mM (1% w/v) lactate with varying Intralipid concentrations (0%–16%) were scanned with both SPIR‐PRESS and long TE PRESS. Common acquisition parameters for all phantom studies included a TR of 7000 ms, 32 averages, and a spectral width of 2564.1 Hz (20 ppm).

#### 2.2.3 In Vivo Animal Studies

Common acquisition parameters for all in vivo studies included a spectral width of 2564.1 Hz (20 ppm), 512 FID points, a TR of 5000 ms, and a CHESS water suppression bandwidth of 350 Hz. The SPIR‐PRESS sequence for all in vivo studies utilized a Gaussian inversion pulse (10.96‐ms duration, 250‐Hz bandwidth). No motion correction or respiratory triggering was applied during the acquisitions. For the glioblastoma brain models, a 3 × 4 × 4‐mm^3^ voxel was placed in the tumor core and acquisition was acquired for 300 transients. As the metabolic profile of the contralateral normal brain region could be influenced by the large tumor, another untreated athymic mouse was also prepared to obtain MRS of normal brain tissue control. Both SPIR‐PRESS (TE = 16.5 ms) and conventional long TE PRESS (TE = 288 ms) sequences were acquired using the standard Bruker quadrature coil. For the UOK161 leg xenograft model, a 5 × 5 × 5‐mm^3^ voxel was placed in the tumor core. SPIR‐PRESS and conventional long TE PRESS acquisitions were performed using the home‐built dual‐tuned coil with the same sequence parameters as the brain model with 60 averages.

#### 2.2.4 Lactate Quantification and Baseline Correction

Consensus linear‐combination modeling was evaluated for spectral quantification. For phantom data, automated quantification was not performed, as the nonstandard, strongly mobile‐lipid matrix could not be reliably simulated to generate a meaningful basis set. For in vivo data, three established software pipelines were tested: Osprey [39], LCModel (via the Osprey interface) [40], and Tarquin [41]. Both Osprey and its LCModel implementation produced highly flexible cubic‐spline baselines that yielded visually acceptable residuals but highly variable and improbable metabolite estimates [42], even with manual phase and referencing adjustments. Tarquin provided a more stable fit but could not accommodate the phase‐inverted lactate peak due to its nonnegativity constraint, leaving a conspicuous residual at 1.3 ppm (Figure S1). These failure modes are consistent with community‐wide findings from the 2016 ISMRM MRS Fitting Challenge [43], which documented poor quantification accuracy for short‐echo, lipid‐contaminated spectra.

Given these documented limitations with automated approaches, lactate was quantified by manual doublet integration after spline‐based baseline correction. FID files were imported into Mestrenova v11.0 (Mestrelab Research) for processing. For in vivo spectra only, a spline‐based “Smooth Segments” routine was used to model the baseline prior to peak integration. Control points were placed on flat spectral regions between 3.67 and 0.45 ppm that were devoid of metabolite signal: the region immediately downfield of the water signal (3.67 ppm), flat zones between NAA and glutamate/glutamine resonances (1.74 ppm), between GABA and glutamine signals (2.7 ppm), and upfield of the mobile lipid tail (0.45 ppm) (Figure S2). These regions were consistent across all mouse spectra. Mestrenova interpolated a cubic spline through these anchor points, and the resulting baseline was subtracted from the spectrum. The lactate doublet was then integrated over a ± 0.06 ‐ppm window centered at 1.33 ppm.

#### 2.2.5 Ex Vivo NMR

For ex vivo analysis, mice were sacrificed by cervical dislocation immediately following the final in vivo MRS scan. The UOK161 leg xenograft tumors were rapidly excised, and the tissue was snap‐frozen in liquid nitrogen within 2 min of harvesting to halt metabolic activity. Metabolites were then extracted from the frozen tissue using the Fan Extraction Method [44]. The resulting aqueous fractions for NMR were lyophilized and stored at −80°C until analysis. For NMR spectroscopy, a polar fraction was dissolved in a 200‐μL deuterium oxide (D2O) solution containing 0.25‐μM sodium 2,2‐dimethyl‐2‐silapentane‐5‐sulfonate‐d₆ (DSS‐d6) as a reference and concentration standard. The experiments were performed at 293 K with a 16.45 T Bruker Avance Neo spectrometer using a 3‐mm inverse triple resonance cryoprobe. Spectra were acquired with a 2‐s acquisition time and a 6‐s recycle delay. Presaturation was used in each case to suppress the residual water signal.

## 3 Results

The SPIR‐PRESS pulse sequence is shown in Figure 1A and consists of a spectrally selective 180° inversion Gaussian pulse inserted before a standard PRESS pulse sequence with VAPOR or CHESS water suppression [45]. The delay time after the inversion pulse (τ) is set to nullify the lipid signal. Relaxation after inversion occurs during the water suppression period (Figure 1B). A short echo time of 16.5 ms is used to maximize the lactate signal.

The T1 nullification method requires a substantial difference in the T1 relaxation time between lipid and lactate. To measure the relaxation difference in a controlled environment, we created two one component phantoms: a 4% w/v lipid emulsion phantom (Intralipid from Sigma, 30% Soybean Oil, 1.2% Egg Yolk Phospholipids, 1.7% Glycerin) and a 1% w/v lactate phantom pH neutralized through titration with sodium hydroxide. The T1 values of lipid and lactate, determined through a three‐parameter exponential fitting, are shown in Figure 2. The T1 of lactate (1387 msec) is more than five times longer than that of lipid (250 msec), potentially enabling the effective detection of negative z magnetization of lactate when the delay time after the inversion pulse (τ) is set to nullify the lipid signal (Figure 1C,D).

To characterize the lipid nulling effect, we analyzed SPIR‐PRESS spectra from the 4% lipid phantom across a range of τ values (Figure 2A). The results show a clear progression from a negative lipid signal (158 msec) through a null point (162 msec) to a positive signal (176 msec), effectively mapping the inversion recovery process and identifying the optimal delay for lipid suppression (Figure 2A). The potential advantages of SPIR‐PRESS over conventional PRESS for lactate quantification are evident in the spectral comparison in Figure 2B. While the PRESS spectrum (bottom row) shows a dominant lipid peak that masks the lactate signal, SPIR‐PRESS (middle row) successfully suppresses the lipid, exposing the lactate doublet at 1.32 ppm. The suppressed signal spectrum (Sp1–Sp2) shows that the inversion pulse's effect is limited to the 0.7‐ to 1.9‐ppm range, underscoring the targeted nature of the band‐selective approach.

We next assessed the quantitative performance of SPIR‐PRESS in differentiating lactate signals from overlapping lipid signals. Phantoms containing lactate and 4% Intralipid were used to compare the relative sensitivity of SPIR‐PRESS and conventional PRESS with a long echo time (TE = 288 ms). Short echo SPIR‐PRESS (Figure 3A) was found to have ~60% increase in sensitivity as measured by the calibration slope compared to conventional PRESS with a 288‐ms TE (Figure 3B). To further evaluate the effectiveness of SPIR‐PRESS in a more physiologically relevant setting, we prepared 1:1 lactate/lipid phantoms containing lactate, NAA, glutamate, choline, and creatine at concentrations mimicking those found in the brain under normal and pathological conditions (lactate [2–20 mM], NAA [5.4–20 mM], choline [1.35–5.35 mM], glutamine [1–4.6 mM], creatine [6–10 mM], pH 7) (Supplementary Table 1) [46, 47, 48]. The delta Akaike information criterion [49] with correction for small sample sizes (ΔAICc) [50] was used to assess whether the regression slopes quantification differed significantly. This approach allows us to compare the potential sensitivity of the two techniques while accounting for our limited sample size.

As shown in Figure 4 and Figure S3, the lactate peak was indistinguishable from the lipid signal when using PRESS with short echo time (TE = 16 ms, Figure 4A) or with intermediate echo time (TE = 90 ms, Figure 4C), which is more reflective of clinical practice. Although both methods quantified lactate with high accuracy (*R* > 0.85), SPIR‐PRESS demonstrated superior performance across multiple metrics, likely due to its shorter echo time. Compared to long‐TE PRESS (Figure 4D), SPIR‐PRESS showed higher linearity (*R* = 0.94 vs. 0.85) and produced a 60% steeper calibration slope, a difference confirmed to be highly significant (ΔAICc = 28.26, *p* < 0.0001) (Figure 4B). This enhanced analytical sensitivity translated to a 41% lower limit of detection at the 95% confidence interval, which fell from 5.82 to 3.40 mM. For NAA, the metabolite peak closest to the inversion pulse, both methods showed equivalent sensitivity (ΔAICc = −3.13). The accuracy of NAA quantitation was significantly reduced when using PRESS with short and intermediate echo times, likely due to the presence of an unsuppressed lipid signal from the allylic carbon (‐CH2‐CH=CH‐) resonating near 2 ppm [51].

We then investigated the impact of varying lipid content (0%–16%) on lactate detection using PRESS and SPIR‐PRESS sequences (Figure 5). High concentrations of both lactate and lipid were used to detect any nonlinearity in the measurements. Both methods clearly detected lactate, as shown by the doublet peaks in Figure 5A, at a fixed lactate concentration of 1%. However, because of the shorter echo time (16.5 ms) used in SPIR‐PRESS, some of the lipid signal was preserved, unlike in PRESS (288 ms), which completely eliminated the lipid signal. Consequently, SPIR‐PRESS showed a constant lactate signal for lower lipid content (< 6%), while PRESS maintained a constant signal across all lipid levels (Figure 5B). This observation suggests that SPIR‐PRESS may be susceptible to baseline shifts in the presence of strong lipid signals, potentially impacting the accuracy of lactate quantification. Despite this limitation, SPIR‐PRESS demonstrated a significantly stronger lactate signal, more than twice that of PRESS, highlighting its potential for sensitive lactate detection in tissues with low to moderate fat content. However, caution should be exercised when using SPIR‐PRESS in tissues with high lipid content, as the strong lipid signal may interfere with accurate lactate quantification.

**FIGURE 6:**
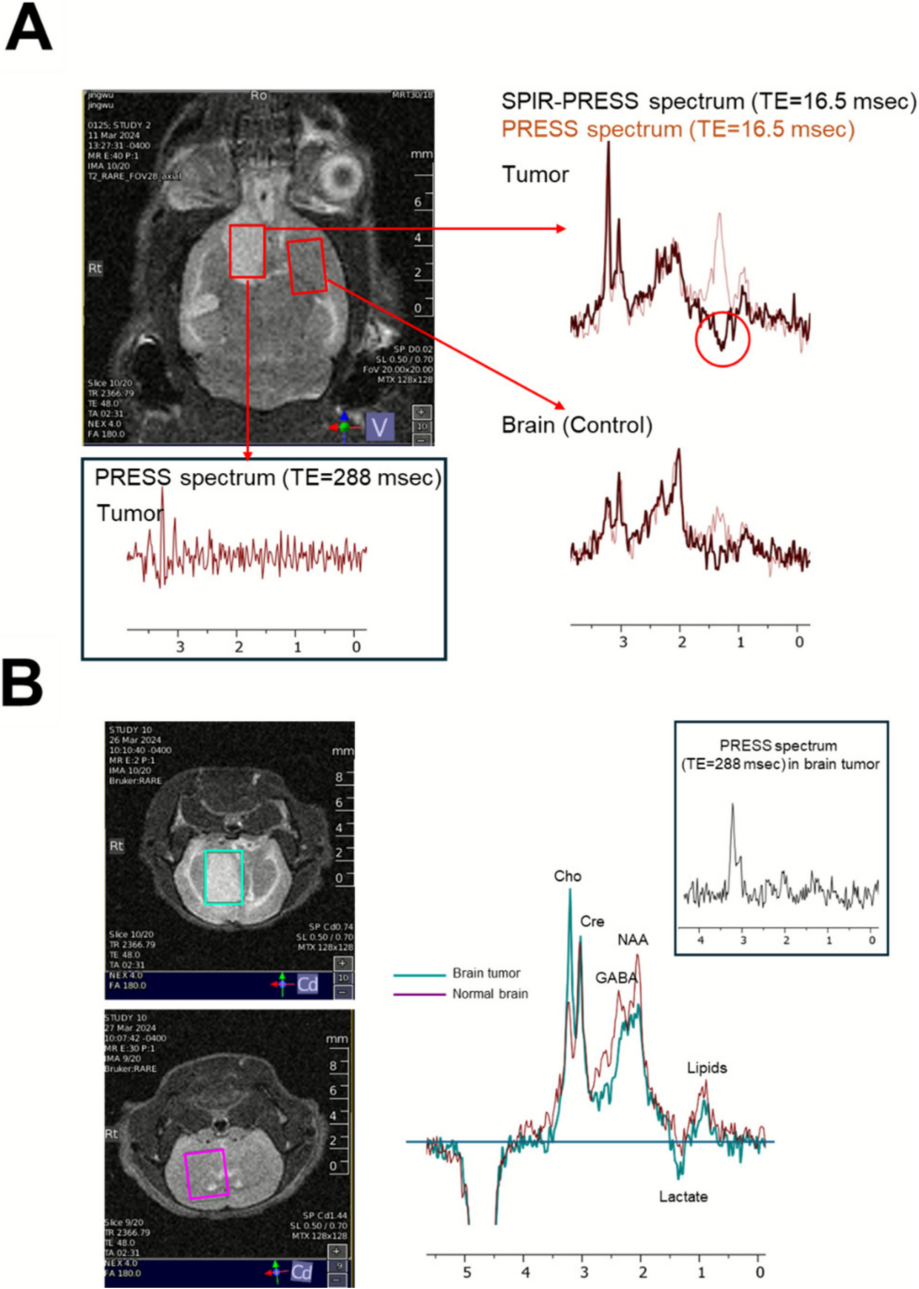
**(A)** Mouse brain with voxels placed in a GBM1 glioma tumor and normal brain at the arrowed sites. The right inset shows overlaid spectra each site: SPIR‐PRESS (dark brown) and short‐TE PRESS (pink). The inverted lactate peak in SPIR‐PRESS spectra, which is obscured by the lipid. The bottom inset shows the corresponding long‐TE PRESS spectrum from the tumor voxel. (**B)** Repeat of the experiment with another mouse using normal brain tissue from a third mouse (pink voxel) for the comparison. The center inset shows the superimposed SPIR‐PRESS spectrum from each mouse. Lactate is present in the mouse bearing the glioma tumor but not in the normal brain tissue of the other mouse. The corresponding long‐TE PRESS from the brain tumor is shown in the right inset.

To validate the SPIR‐PRESS method in vivo, we compared its performance to that of conventional long TE PRESS in a mouse brain tumor model (*N* = 3). In the first mouse, 2 × 3 × 3 mm voxels were placed in the tumor and contralateral normal brain (Figure 6A). The long TE (288 ms) PRESS tumor spectrum had poor sensitivity due to substantial T2 relaxation at long echo times, with only faint total choline and creatine peaks visible (Figure 6A). In contrast, the short TE (16.5 ms) SPIR‐PRESS tumor spectrum clearly showed distinct total choline, creatine, and NAA peaks. An inverted lactate peak at 1.3 ppm provided proof of the presence of lactate in the tumor (Figure 6A, dark brown). Except for the negative lactate peak, the SPIR‐PRESS spectrum is nearly identical to the short TE PRESS spectrum without the inversion pulse (red), confirming that the measurement of other metabolites was not affected by the inversion pulse. These findings were replicated in two separate mice by comparing a tumor‐bearing mouse to a separate healthy control mouse without a contralateral voxel (Figure 6B).

In the contralateral negative control voxel, a peak at 1.3 ppm is present in the short TE PRESS spectrum that could be confused for lactate (Figure 6A). However, this peak is absent in the SPIR‐PRESS spectrum, indicating that it arises from lipids rather than lactate. Given that lactate in normal brain grey matter is only 0.5 mM, this peak is almost certainly lipid, indicating successful nulling of the lipid signal at 1.3 ppm by the inversion pulse. A separate large IDH mutant glioma (Figure S4A) yielded a minimal lactate doublet with SPIR‐PRESS (Figure S4B) and no lactate with long‐TE PRESS (Figure S4C), confirming that the approach can separate cases with elevated lactate from those with low lactate signal. These findings underscore the capability of SPIR‐PRESS to accurately identify key tumor metabolites, including lactate, without interference from lipid signals that could be misinterpreted in the short TE PRESS spectrum. This represents a significant improvement over the conventional long TE PRESS, which failed to detect lactate and whose spectrum is predominantly characterized by noise.

**FIGURE 7:**
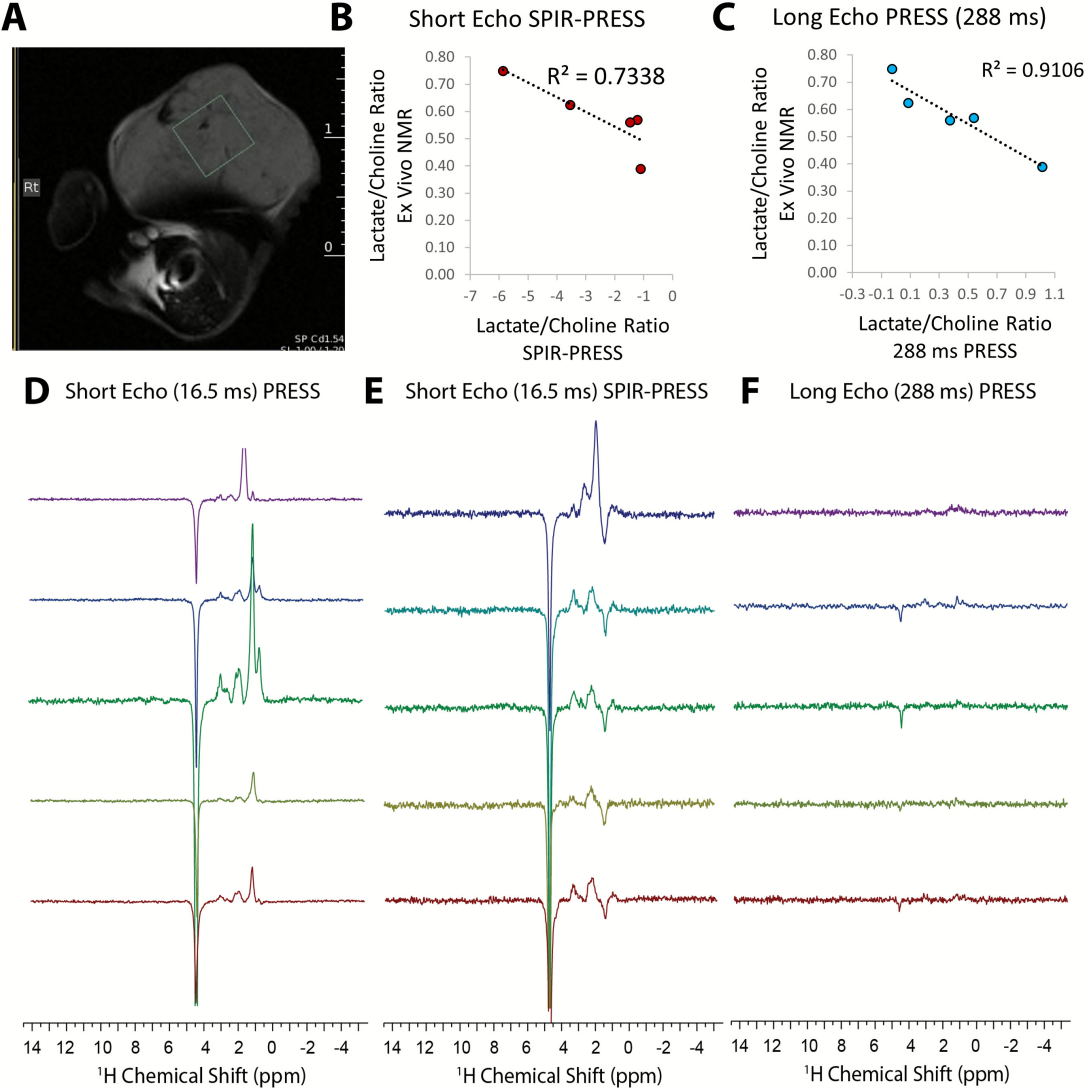
UOK161 leg xenograft tumor analysis: (A) T2‐weighted RARE image showing voxel placement in the tumor. (B) Correlation between lactate/total choline ratios from short‐echo SPIR‐PRESS and ex vivo NMR measurements (*R* = 0.86). C Correlation between lactate/total choline ratios from long‐echo PRESS (288 ms) and ex vivo NMR measurements (*R* = 0.95). (D–F) Short‐TE PRESS (16.5 ms). (E) Short‐TE SPIR‐PRESS (16.5 ms). (F) Long‐TE PRESS (288 ms).

To further challenge the SPIR‐PRESS method, we applied it to a UOK161 renal clear cell carcinoma leg xenograft, a model known for its extremely high lipid content (Figure 7A). Representative spectra from this model are shown in Figure 7D–F. In the conventional short‐TE PRESS spectrum (Figure 7D), a massive lipid signal at 1.3 ppm completely overwhelmed the spectrum, making it impossible to resolve any lactate signal. The conventional long‐TE PRESS acquisition (Figure 7F) resulted in a spectrum with weak metabolite signals, as both lipids and metabolites were attenuated by T2 relaxation. In stark contrast, the SPIR‐PRESS spectrum (Figure 7E) shows effective nulling of the lipid signal, revealing a clear, inverted lactate doublet at 1.3 ppm. Notably, SPIR‐PRESS retains sensitivity to other key metabolites, with the total choline peak at 3.2 ppm remaining clearly visible.

To validate these in vivo findings, lactate/total choline ratios from both SPIR‐PRESS and long‐TE PRESS were correlated against measurements from ex vivo NMR of the harvested tumors. Both methods showed a strong correlation with the ex vivo results, with *r* values of 0.86 for SPIR‐PRESS (Figure 7B) and 0.95 for long‐TE PRESS (Figure 7C). This confirms that even in a challenging, high‐lipid environment where conventional methods fail to provide a full metabolic profile, SPIR‐PRESS can successfully detect lactate while preserving other important metabolite signals.

## 4 Discussion

Detecting tissue hypoxia and metabolic dysregulation is crucial for diagnosing and monitoring numerous diseases, making the accurate in vivo measurement of lactate—a hallmark of this state—a primary goal for clinical spectroscopy. In neuro‐oncology, elevated lactate is a powerful indicator of tumor aggressiveness and is correlated with higher tumor grades and a poorer prognosis [52]. Beyond cancer, its presence serves as a direct indicator of oxygen starvation and neuronal distress in acute neurological events such as stroke and traumatic brain injury. In conditions like mitochondrial disorders, MRS can detect elevated cerebral lactate even when conventional imaging shows no structural abnormalities. Crucially, the concentration of lactate is not just a binary indicator of presence or absence; it is potentially a quantitative marker that can correlate with disease severity [10, 52, 53], creating an urgent need for methods that can measure it accurately.

The primary obstacle to the reliable detection of lactate via ^1^H‐MRS is the severe spectral overlap of its target methyl doublet at 1.33 ppm with strong, broad signals from other molecules. Resonances from the methylene (–CH_2_–) groups of mobile lipids appear at a near‐identical position. In many pathological conditions where lactate is elevated, such as high‐grade tumors with necrotic cores, lipid levels are also significantly increased, leading to a massive lipid signal that can completely obscure the much smaller lactate doublet, making even the simple identification of lactate exceptionally difficult. In these challenging, lipid‐rich environments, SPIR‐PRESS has potential as a simple, fast, and robust method for the semiquantitative determination of relative lactate concentrations, providing a significant improvement over conventional PRESS at both long and short echo times.

The phantom experiments confirmed that SPIR‐PRESS can accurately distinguish lactate from lipid signals, with a potentially lower limit of detection. Furthermore, our in vivo results in a mouse brain tumor model verified the ability of SPIR‐PRESS to detect lactate in tumors while suppressing lipid signals that could be misinterpreted in short TE PRESS spectra. The clear detection of other key tumor metabolites, such as total choline, creatine, and NAA, highlights the method's primary advantage: It maintains sensitivity to the full metabolite profile. This is crucial for diagnostic and monitoring purposes not only in brain tumors but across a range of pathologies, including stroke, ischemia, and mitochondrial disorders where comprehensive metabolic assessment is vital.

There are nonetheless a number of drawbacks to the SPIR‐PRESS method, which can be categorized as either intrinsic to the technique or specific to the current implementation. The primary intrinsic limitations relate to the fundamental dependence on T_1_‐based nulling. The success of the method requires an accurate lipid T_1_ relaxation time to completely null the lipid signal. This is complicated by the fact that mobile lipids are not a single chemical species but a mixture with a distribution of T_1_ values; a single inversion time cannot perfectly null all components simultaneously. Furthermore, the accuracy of the null point is susceptible to B_1_ (RF field) and B_0_ (static field) inhomogeneities, which can cause incomplete inversion across the voxel and leave residual lipid signal. This effect can be mitigated by adiabatic inversion pulses as mentioned above. The current implementation uses a simple inversion sequence to minimize the echo time for maximum sensitivity. In mice, a single inversion time was sufficient to null the lipid signal (Figure 6 and S4). In a clinical situation, there is also a moderate but measurable interpatient variation of the T1 relaxation rate that could be accounted for by a double or multiple inversion recovery sequence [54]. Similarly, sLASER can be used instead of PRESS to improve localization and phase coherence, although a recent study found both sequences perform similarly at 3 T for short echo times [55]. Finally, the lactate signal itself is also attenuated slightly by T_1_ relaxation after the inversion pulse, an inherent trade‐off of the design.

Other limitations are specific to the current study's design and could be addressed in future work. The narrow selective pulse used here does not invert the α‐olefenic lipid peaks at 2.2 and 2.5 ppm, although the problem is highly exaggerated in the phantom by the very high content of PUFA in soybean oil (58%) compared to human tissue (2%) [56]. More fundamentally, the current implementation possesses the limitations inherent to all short echo time spectroscopy techniques [57]. Glutamate and glutamine in particular are not resolved from each other or resolved fully from the NAA peak. Automated quantification was also challenging because the selective pulse only suppresses part of the macromolecule baseline; this could be addressed by acquiring a separate, metabolite‐nulled macromolecule spectrum for use in the fitting basis set at the expense of acquisition time [58, 59]. Despite these limitations, SPIR‐PRESS represents a promising approach for detecting lactate in brain tumors with improved sensitivity and specificity compared to conventional long TE PRESS, enabling more accurate assessment of the role of lactate in tumor metabolism and mitochondrial disease. Further validation is warranted to establish its possible clinical utility.

## Author Contributions

Conceptualization: S.K., J.R.B; funding acquisition: M.C.K., W.M.L.; methodology: S.K., J.R.B; resources: O.K., P.L., J.W.; writing—original draft: S.K., J.R.B.; writing—review and editing: J.R.B; investigation: S.K., J.R.B, D.R.C., J.M., O.Y., Y.K., Y.S., K.Y.

## Funding

This work was supported by intramural funds provided by the Center for Cancer Research, National Cancer Institute of the National Institutes of Health (ZIC BC011932).

## Supporting information


**Figure S1:** Tarquin linear‐combination fit of an in vivo SPIR‐PRESS spectrum illustrating limitations of automated modeling for phase‐inverted lactate: The nonnegativity constraint prevents fitting of the inverted lactate doublet at ~1.33 ppm, leaving a prominent residual, and the accompanying lipid signals flanking lactate are poorly modeled; the lactate resonance at ~4.1 ppm is likewise not fit.
**Figure S2:** Spline‐based baseline correction strategy for in vivo SPIR‐PRESS quantification (UOK161 renal clear cell carcinoma leg xenograft). To accommodate the phase‐inverted lactate peak, a cubic spline baseline was modeled using control points placed in spectral regions determined to be devoid of significant metabolite or lipid signals. Anchor points were consistently placed at approximately 3.67 (downfield of water), 2.7, 1.74, and 0.45 ppm (upfield of the mobile lipid tail) prior to subtraction and peak integration.
**Figure S3:** Full short‐TE SPIR‐PRESS spectra from the complex phantom used in Figure 4.
**Figure S4:** In vivo spectra from a large IDH mutant glioma with reduced lactate levels. A, T2‐weighted MR image showing voxel placement in the tumor. B, Overlaid short‐TE PRESS (grey) and SPIR‐PRESS (red) spectra; the SPIR‐PRESS spectrum shows a lactate signal that is less than WT IDH after baseline correction, but not zero. C, Corresponding long‐TE PRESS spectrum, showing no distinct lactate signal.
**Table S1:** Metabolite concentration for the mixed metabolite phantom used in Figure 4.

## Data Availability

The data supporting the findings of this study are openly available in Harvard Dataverse at https://doi.org/10.7910/DVN/IGSHZW.
