# Supplementary material for: Band‐Selective IR PRESS for Brain Tumor Spectroscopy Allows Robust Detection of Lactate
Source: NMR Biomed. 2026 Feb 2;39(3):e70220. doi: 10.1002/nbm.70220 (PMC12863986; doi:10.1002/nbm.70220)
Supplement: Supplementary file 1 — Figure S1: Tarquin linear‐combination fit of an in vivo SPIR‐PRESS spectrum illustrating limitations of automated modeling for phase‐inverted lactate: The nonnegativity constraint prevents fitting of the inverted lactate doublet at ~1.33 ppm, leaving a prominent residual, and the accompanying lipid signals flanking lactate are poorly modeled; the lactate resonance at ~4.1 ppm is likewise not fit. Figure S2: Spline‐based baseline correction strategy for in vivo SPIR‐PRESS quantification (UOK161 renal clear cell carcinoma leg xenograft). To accommodate the phase‐inverted lactate peak, a cubic spline baseline was modeled using control points placed in spectral regions determined to be devoid of significant metabolite or lipid signals. Anchor points were consistently placed at approximately 3.67 (downfield of water), 2.7, 1.74, and 0.45 ppm (upfield of the mobile lipid tail) prior to subtraction and peak integration. Figure S3: Full short‐TE SPIR‐PRESS spectra from the complex phantom used in Figure 4. Figure S4: In vivo spectra from a large IDH mutant glioma with reduced lactate levels. A, T2‐weighted MR image showing voxel placement in the tumor. B, Overlaid short‐TE PRESS (grey) and SPIR‐PRESS (red) spectra; the SPIR‐PRESS spectrum shows a lactate signal that is less than WT IDH after baseline correction, but not zero. C, Corresponding long‐TE PRESS spectrum, showing no distinct lactate signal. Table S1: Metabolite concentration for the mixed metabolite phantom used in Figure 4. [file NBM-39-e70220-s001.docx]

**Band-selective IR PRESS for brain tumor spectroscopy allows robust detection of lactate**

Shun Kishimoto,^1,2^ Daniel R. Crooks,^2^ Peng Lu, ^3^ Yuki Shibata,^2^ Olga Kim,^3^ Jeeva Munasinghe,^4^ Otowa Yasunori,^1^ Yamashita Kota,^1^ Kazutoshi Yamamoto,^1^ W. Marston Linehan,^2^ Jing Wu,^3^ Murali C Krishna,^1^ Jeffrey R Brender^1,5^

^1^ Radiation Biology Branch, Center for Cancer Research, National Cancer Institute, National Institutes of Health, Bethesda, MD 20892, USA

^2^ Urologic Oncology Branch, Center for Cancer Research, National Cancer Institute, National Institutes of Health, Bethesda, MD 20892, USA

^3^ Neuro-Oncology Branch, Center for Cancer Research, National Cancer Institute, National Institutes of Health, Bethesda, MD 20892, USA

^4^ Laboratory of Functional and Molecular Imaging, National Institute of Neurological Disorders and Stroke, National Institutes of Health, Bethesda, MD 20892, USA

^5^ Molecular Imaging Branch, National Cancer Institute, National Institutes of Health, Bethesda, MD 20892, USA

Correspondence: Jeffrey R. Brender, jeffrey.brender@nih.gov

**Keywords:** Magnetic Resonance Spectroscopy, Lactate, Lipid, Inversion Recovery

**Abbreviations:** SPIR-PRESS **:** Spectral Presaturation with Inversion Recovery, RARE: Rapid Acquisition with Relaxation Enhancement**,** CHESS: Chemical Shift Selective, ΔAICc: delta Akaike Information Criterion with correction for small sample sizes, NAA: N-acetyl aspartate


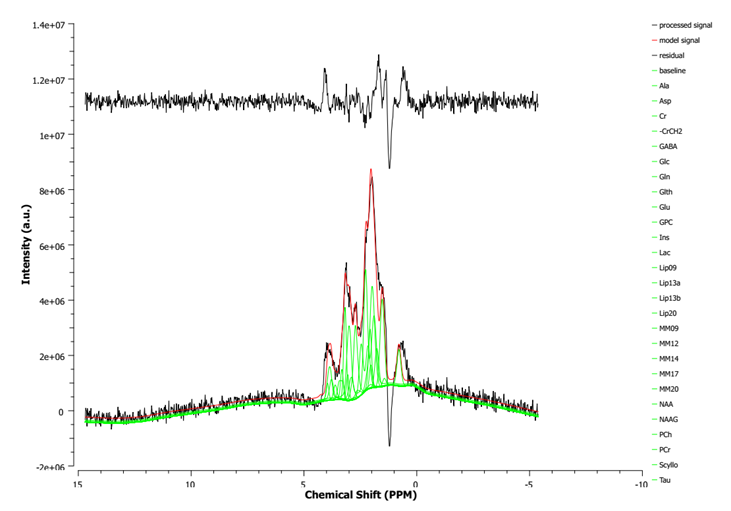


**Figure S1.** Tarquin linear‑combination fit of an in vivo SPIR‑PRESS spectrum illustrating limitations of automated modeling for phase‑inverted lactate: the non‑negativity constraint prevents fitting of the inverted lactate doublet at ~1.33 ppm, leaving a prominent residual, and the accompanying lipid signals flanking lactate are poorly modeled; the lactate resonance at ~4.1 ppm is likewise not fit.


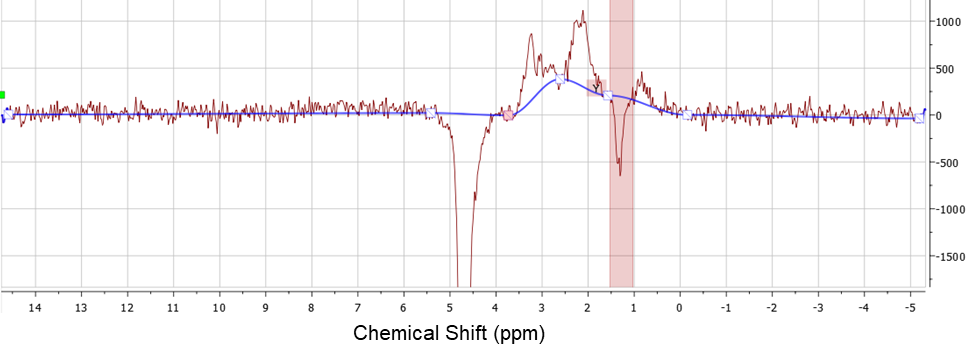


**Figure S2**: Spline-based baseline correction strategy for in vivo SPIR-PRESS quantification (UOK161 renal clear cell carcinoma leg xenograft). To accommodate the phase-inverted lactate peak, a cubic spline baseline was modeled using control points placed in spectral regions determined to be devoid of significant metabolite or lipid signals. Anchor points were consistently placed at approximately 3.67 ppm (downfield of water), 2.7 ppm, 1.74 ppm, and 0.45 ppm (upfield of the mobile lipid tail) prior to subtraction and peak integration.

**
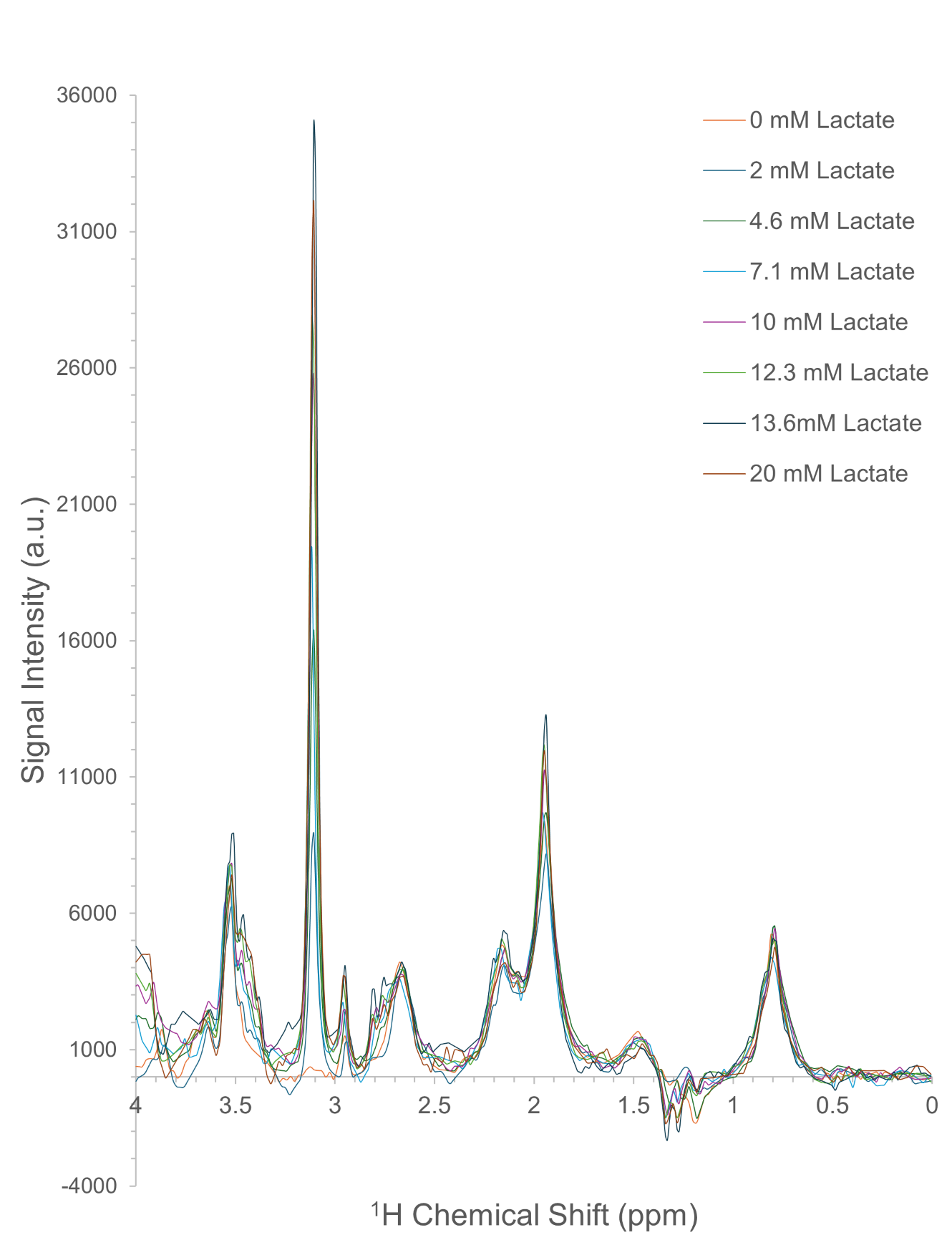
**

**Figure S3.** Full short‑TE SPIR‑PRESS spectra from the complex phantom used in Figure 4

**
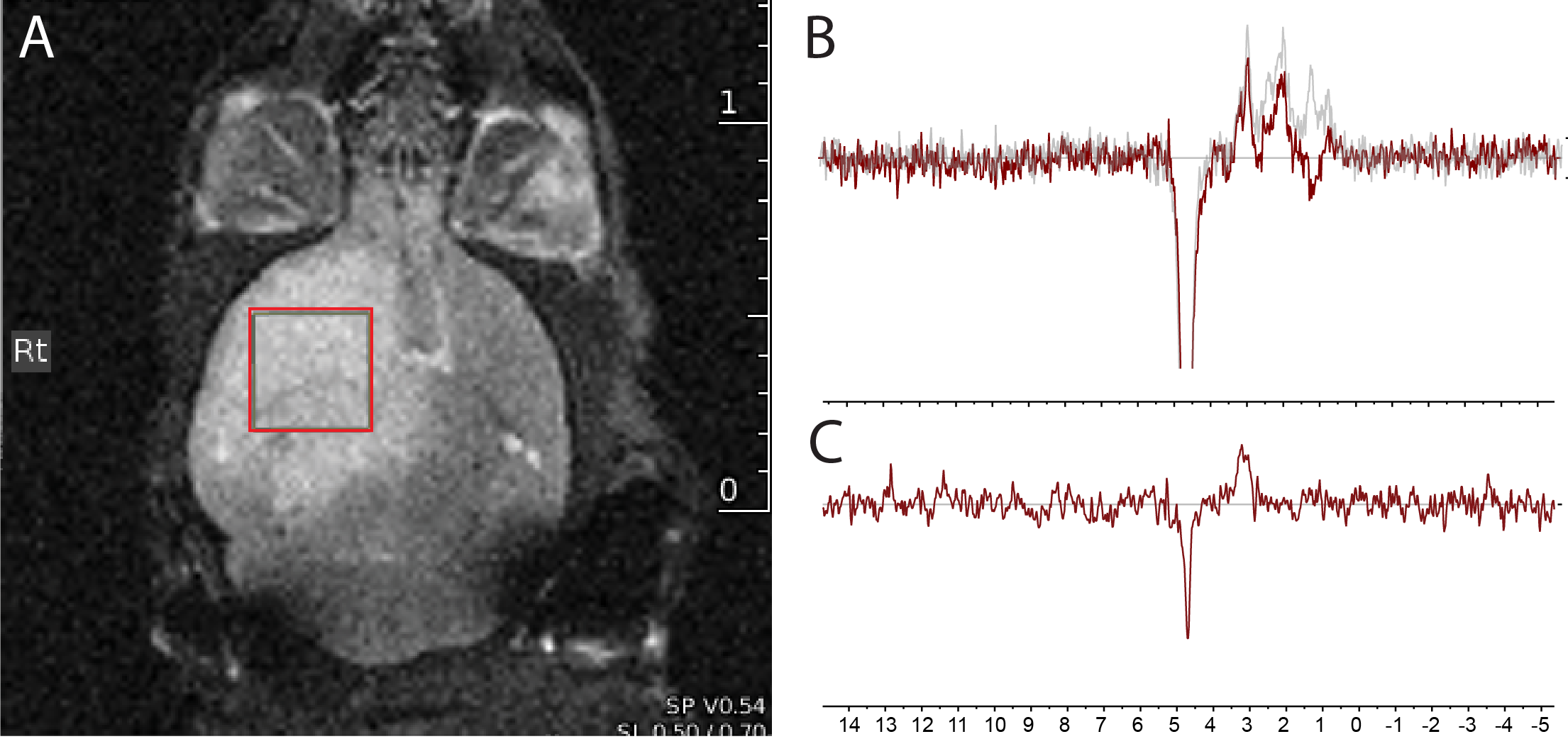
**

**Figure S4:** In vivo spectra from a large IDH mutant glioma with reduced lactate levels. A, T2-weighted MR image showing voxel placement in the tumor. B, Overlaid short-TE PRESS (grey) and SPIR-PRESS (red) spectra; the SPIR-PRESS spectrum shows a lactate signal that is less than WT IDH after baseline correction, but not zero. C, Corresponding long-TE PRESS spectrum, showing no distinct lactate signal.

| Sample | Lactate (mM) | Lipid (mM) | NAA (mM) | Choline (mM) | Glutamine (mM) | Creatine (mM) |
| --- | --- | --- | --- | --- | --- | --- |
| 1 | 2.0 | 2.0 | 5.4 | 1.35 | 1 | 6 |
| 2 | 4.6 | 4.6 | 7.1 | 2.02 | 1.67 | 6.60 |
| 3 | 7.1 | 7.1 | 9.3 | 2.68 | 2.33 | 7.20 |
| 4 | 10.0 | 10.0 | 11.4 | 3.35 | 3.00 | 7.80 |
| 5 | 12.3 | 12.3 | 13.6 | 4.02 | 3.67 | 8.40 |
| 6 | 13.6 | 13.6 | 15.7 | 4.68 | 4.33 | 9.00 |
| 7 | 20.0 | 20.0 | 20.0 | 5.35 | 5.00 | 9.60 |

**Supplementary Table 1:** Metabolite concentration for the mixed metabolite phantom used in Figure 4.
